# Self-Initiated Actions Under Different Choice Architectures Affect Framing and Target Evaluation Even Without Verbal Manipulation

**DOI:** 10.3389/fpsyg.2020.01449

**Published:** 2020-07-14

**Authors:** Yutaro Onuki, Hidehito Honda, Kazuhiro Ueda

**Affiliations:** ^1^Graduate School of Arts and Sciences, The University of Tokyo, Tokyo, Japan; ^2^Japan Society for the Promotion of Science (JSPS), Tokyo, Japan; ^3^Faculty of Psychology, Otemon Gakuin University, Osaka, Japan

**Keywords:** judgment, framing effect, choice architecture, self-initiated action, evaluation

## Abstract

Logically equivalent but different descriptions (i.e., manipulation of verbal expressions) affect decision-making in a phenomenon known as the *framing effect*. A choice architecture changes decision-makers’ actions, which in turn create different frames, but little is known about whether the frame created by their action can change their judgments. We examined whether self-initiated action induced by a choice architecture changed evaluations. In two experimental studies (*N* = 271), we found that self-initiated actions whose final goal was completely the same and for which no verbal expressions were manipulated led to different evaluations. In particular, we found that a difference in the placement of rewards, which required participants to behave differently, changed their ratings of satisfaction with the rewards. This study provides evidence that the framing effect can occur without verbal manipulation. This finding advances our understanding of how participants’ actions lead to different evaluations.

## Introduction

A *framing effect* occurs when logically equivalent but different descriptions lead to different decisions (Tversky and Kahneman, 1981). Here is an example by Tversky and Kahneman (1981): Respondents see the sentence “The US is preparing for the outbreak of an Asian disease, which is expected to kill 600 people. Two alternative treatments are proposed for combating the disease. Experts estimate the consequences of treatments, and you have to choose one treatment.” In a positive frame, treatment A is “To save 200 lives,” and treatment B is “1/3 chance of saving all 600 people but 2/3 possibility of saving no one.” In a negative frame, treatment C is “400 people will die,” and treatment D is “1/3 chance that no people will die but 2/3 probability that all 600 people will die.” Note that treatment A corresponds to treatment C. Likewise, treatment B corresponds to treatment D. In Tversky and Kahneman’s study (1981), 72% of the participants chose treatment A in the positive frame, but 78% of them chose treatment D in the negative frame. That is, the participants’ decision-making changed depending on how the options were described. Framing effects also happen in consumers’ behaviors such as online shopping (Jin et al., 2017), along with evaluation and purchase intention (Cheng et al., 2014). Thus, framing effects are closely tied to our lives. Although various framing effects have been reported (Levin et al., 1998), in most cases, such effects have been generated by manipulations of verbal expressions.

A previous study showed that a choice architecture changes people’s actions, which in turn create different frames (Sher and McKenzie, 2006). “Choice architecture” refers to the environment of decision-making and the presentation design of the information about choices (Thaler et al., 2010). Imagine the following situation. There are two identical glasses with 500-ml capacity presented in one of the following two situations.

Situation X: Glass A contains 500 ml of water and Glass B 0 ml of water. You are asked, “Please adjust the amount of water in Glass A to 250 ml.”

Situation Y: Glass A contains 0 ml of water and Glass B 500 ml of water. You are asked, “Please adjust the amount of water in Glass A to 250 ml.”

These two situations are completely the same in that your goal is to adjust the amount of water in Glass A to 250 ml. However, the environment (Glass A contains 500 ml or 0 ml of water) and the self-initiated action for Glass A differ: “Removing water into another glass” or “Pouring water from another glass.” A previous finding of Sher and McKenzie (2006) suggested that different actions could be associated with different frames: Glass A in Situation X, asking people to “remove water,” tended to be described as “half empty.” In contrast, Glass A in Situation Y, asking people to “pour in water,” tended to be described as “half full.” This finding indicated that self-initiated action produces different frames for Glass A, which contains 250 ml of water in all cases (i.e., “half empty” or “half full”). It has not, however, been shown whether the different frames that are created by decision-makers’ actions also change their judgments. Based on this previous study, we predicted that the different frames, in all of which Glass A contains 250 ml of water, would affect people’s feelings regarding the amount of water it contains: In Situation X, people may find the amount of water to be “insufficient.” Contrariwise, in Situation Y, people may feel it to be “sufficient.” Thus, we posited the hypothesis of a new type of framing effect, whereby the self-initiated action may change the frame and decision-makers’ evaluations. Note that in the above two Situations X and Y, the presented instruction is the same (“Please adjust the amount of water in Glass A to 250 ml.”). That is, if the framing effect occurs with this procedure, it is not due to verbal manipulation. As in this procedure, we predicted that although the instruction is the same, the choice architecture will implicitly construct different self-initiated actions and different frames, leading to different psychological feelings and evaluations. In this study, we investigated whether the self-initiated actions elicited by the choice architecture changed the frame and decision-makers’ evaluations.

## Experimental Study

The protocols of the following experiments conformed to the Declaration of Helsinki and were approved by the Ethics Review Committee for Experimental Research with Human Subjects at the University of Tokyo.

### Experiment 1

**FIGURE 1:**
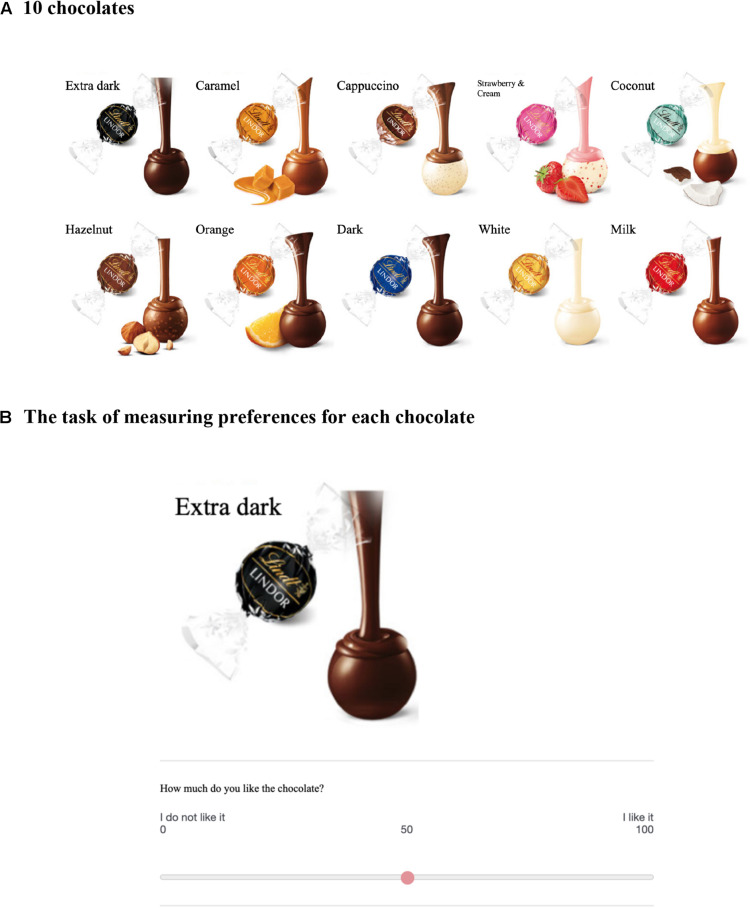
Panel **(A)** shows 10 types of chocolates, and panel **(B)** shows the task of measuring preference for each chocolate. Participants were asked their preference for each of the 10 types of chocolate.

**FIGURE 2:**
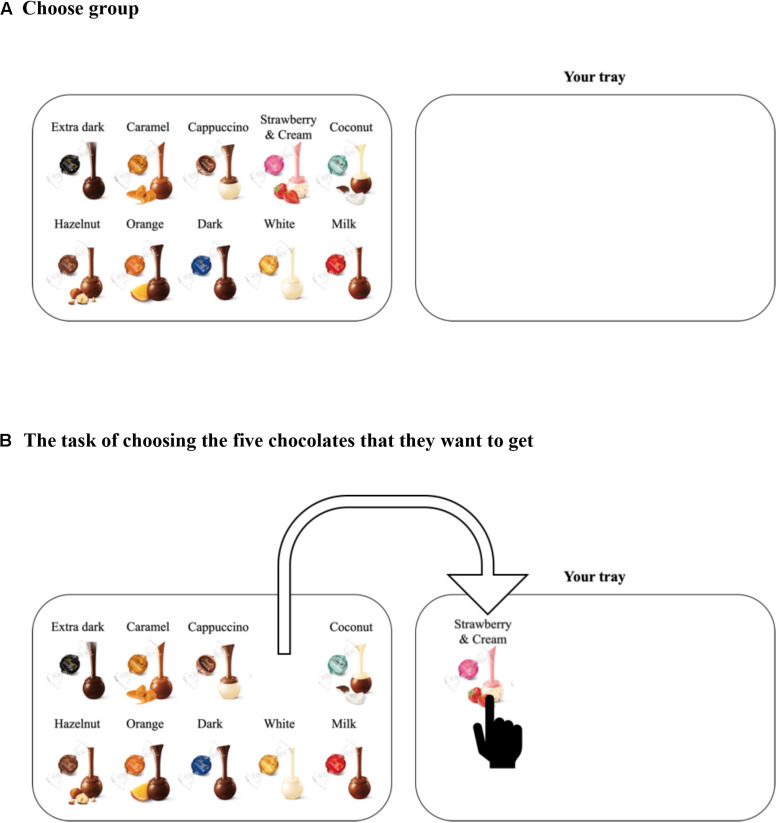
Panel **(A)** shows a screenshot of the stimulus for the choose group. Panel **(B)** shows the situation where a participant dragged the picture of the chocolate from a tray to her/his tray.

**FIGURE 3:**
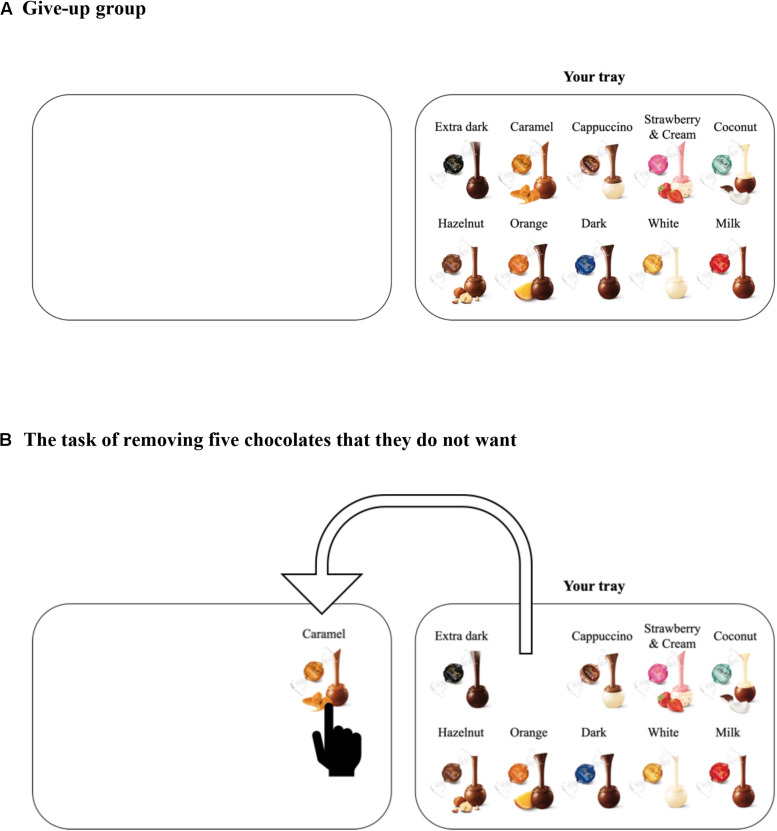
Panel **(A)** shows a screenshot of the stimulus for the give-up group. Panel **(B)** shows the situation where a participant dragged the picture of the chocolate from her/his tray to another tray.

We examined whether self-initiated actions elicited by the choice architecture could yield different evaluations. Participants were instructed, “Please choose 5 of the 10 chocolates you want” (Figure 1). Here, the default position of the 10 chocolates differs between the two situations (Figures 2A, 3A). When their own tray is empty beforehand, participants might think that they could receive five chocolates (Figures 2A,B). In contrast, when their own tray has 10 chocolates beforehand, they might think that they have to “give up” five chocolates (Figures 3A,B). We obtained copyright permissions to use and publish the figures of a product (Lindt Lindor) from LINDT & SPRUNGLI JAPAN. We predicted that the different default positions of the 10 chocolates will induce different self-initiated actions (choose or give-up). Moreover, the self-initiated action will implicitly lead to different frames, which will affect satisfaction with the five chocolates. In particular, the participants may be more satisfied with five chocolates when their own tray is empty (choose group) than when their own tray has 10 chocolates beforehand (give-up group). We note that the participants are given the same verbal instructions and can obtain the same amount of rewards as a result. Previous framing studies typically used positive or negative frame descriptions. In this study, however, the decision-makers are not provided with specific frame descriptions. The differences lie only in the different default positions of the 10 chocolates and the different self-initiated actions.

#### Participants

A total of 223 participants (*M*_*age*_ = 44.25, *SD*_*age*_ = 8.05, *Men* = 124, and *Women* = 99) were recruited from Rakuten Insight^1^ and randomly assigned to one of the two groups (choose or give-up). We conducted the experiment using GUI in Qualtrics^2^. We used the program G^∗^Power (Version 3.1.9.3; Faul et al., 2007) to conduct a power analysis. As a meta-analysis of the framing effect showed that the mean effect size of framing was *d* = 0.329 (Kühberger, 1998; nearly 30,000 single subjects in the meta-analysis), we set the effect size of the power analysis at *d* = 0.329. Moreover, we used a one-tailed analysis because we had hypothesized that the choose group would be more satisfied with the five chocolates than the give-up group. The analysis indicated that a sample size of approximately 115 participants would be necessary for the study to have a detection power of 80% and α = 0.05. Thus, we tried to recruit around 115 participants for each group.

#### Task, Stimulus, and Procedure

First, the participants performed an irrelevant task for 20 min. In the irrelevant task, they guessed which of two city names presented at the same time on their computer screen had the larger population. After the irrelevant task, they were shown 10 different chocolates (Figure 1A) and asked their preference for each chocolate on a computer screen (Figure 1B). They were asked “How much do you like the chocolate?” for each of 10 types of chocolate and answered on a scale labeled “I do not like it” on the far left and “I like it” on the far right. This rating scale contained 101 points. Participants were then randomly assigned to the choose group (*n* = 113; Figure 2A) or give-up group (*n* = 110; Figure 3A). Participants were presented with the following instruction: “Assume that you will get five out of the 10 chocolates as a reward for answering the precedent questions. Please put five chocolates on your tray.” That is, the participants in the choose group were asked to choose five chocolates and drag them *to their tray* (Figure 2B), whereas the participants in the give-up group chose five chocolates and dragged them *from their tray* (Figure 3B). The self-initiated action (choose or give-up) differed depending on the default position of the 10 chocolates. Since Experiment 1 was conducted in the form of a Web survey, participants knew that they would not actually get the chocolates. After the choice of five chocolates, they answered a question; “If you could get five chocolates as a reward for answering the former questions, how much would you be satisfied with the five chocolates?,” where “former questions” refer to the preceding irrelevant tasks. They answered the question using a scale labeled “I’m not satisfied” on the far left and “I’m satisfied” on the far right on the computer screen. This rating was recorded with 101 points.

#### Results

**FIGURE 4:**
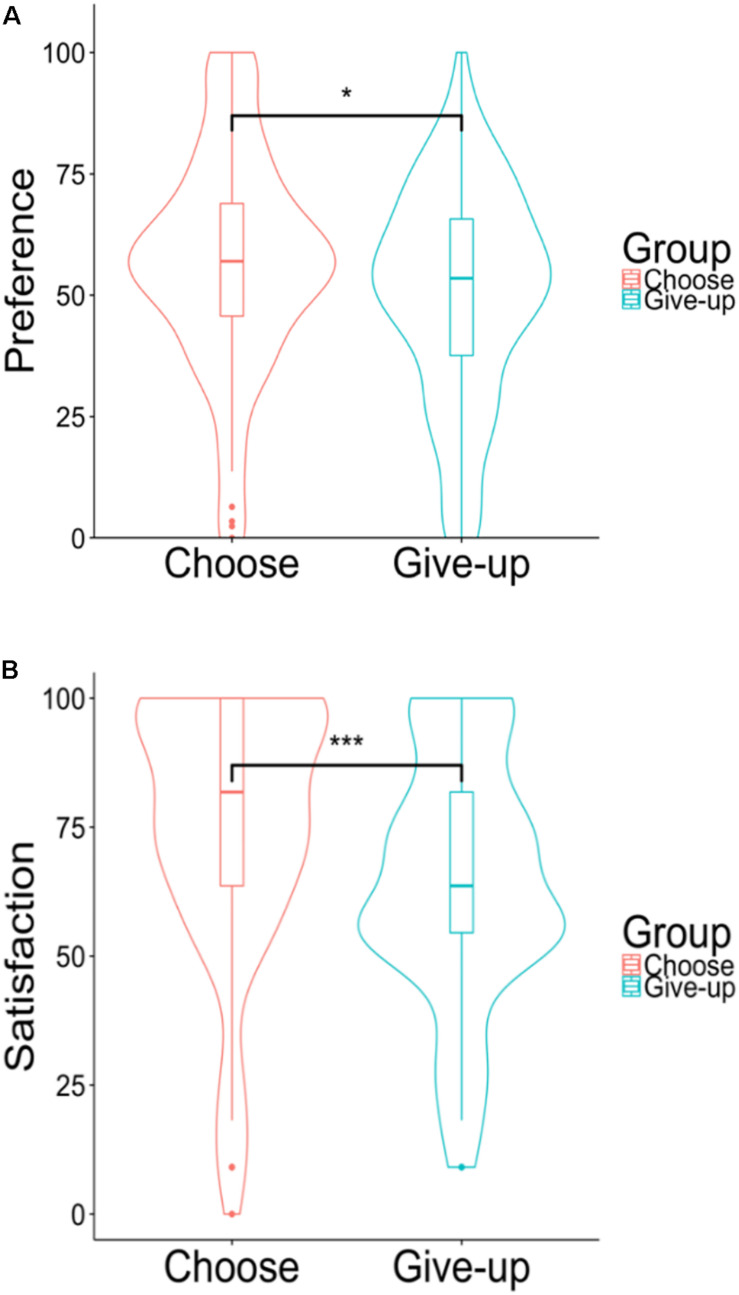
**(A)** Violin plots of the preferences for the 10 chocolates. **(B)** Violin plots of the hypothetical satisfaction with the five chocolates. *p < 0.05, ***p < 0.001.

Figure 4 shows the distribution (violin plot) of the preference for the chocolates and satisfaction with the five chocolates for the two groups. In the preference for the 10 chocolates, there was a significant difference between the choose group (*Mean* = 56.44, *SD* = 22.50) and the give-up group [*Mean* = 50.46, *SD* = 22.15; *t*(221) = 1.99, *p* = 0.047, and *d* = 0.27]. The satisfaction with the five chocolates for the choose group (*Mean* = 75.62, *SD* = 25.37) was significantly higher than for the give-up group [*Mean* = 64.05, *SD* = 23.76; *t*(221) = 3.52, *p* < 0.001, and *d* = 0.46]. We performed a multiple regression analysis to check whether self-initiated action still affected satisfaction by controlling for the preference for the 10 chocolates. Specifically we regressed the rating of satisfaction on two variables, the group (i.e., choose or give-up, a dummy variable) and the preference for the 10 chocolates. Table 1 shows the results of the multiple regression analysis. The coefficients of preference for the 10 chocolates and of group were significant.

**TABLE 1:** Results of multiple regression analysis of satisfaction with the rewards by two factors.

**Relevant factors**	**β**	***SE***	***t*-value**	***p*-value**
Intercept	42.827	4.068	10.526	*p* < 0.001***
Group (choose or give-up)	–8.103	2.829	–2.864	0.004**
Preference for the 10 chocolates	0.581	0.063	9.212	*p* < 0.001***

***p < 0.001, **p < 0.01.

**FIGURE 5:**
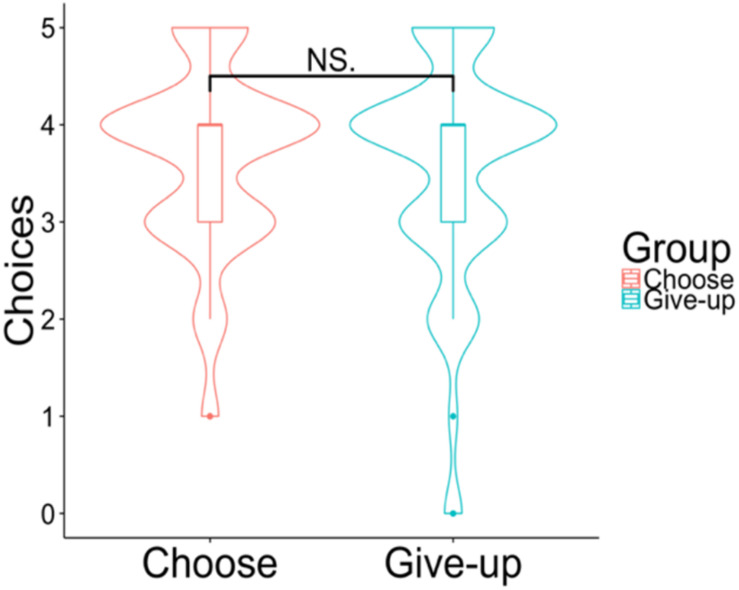
Violin plots of the choices. The *y*-axis shows how many chocolates the participants selected among their top five preferences. *NS p* > 0.05.

We analyzed whether self-initiated action affected choice consistency. If participants selected the five reward chocolates for which the preference was low, the choice might reduce their satisfaction with the reward. Thus, we analyzed whether the participants actually selected the chocolates for which they had higher preference over those for which they had a lower preference. In particular, we investigated whether participants actually selected the top five preferred chocolates as the reward. Figure 5 shows the distribution of how many chocolates the participants actually chose among their favorite chocolates from the top five. For choices, no significant difference was found between the choose group (*Mean* = 3.64, *SD* = 0.95) and the give-up group [*Mean* = 3.53, *SD* = 1.17; *t*(210) = 0.77, *p* = 0.444, and *d* = 0.12].

#### Discussion

We found that self-initiated action generated by the default position of 10 chocolates affected satisfaction with the five chocolates. Unexpectedly, the prior preference for the 10 chocolates was significantly different between the choose and give-up groups, even though the participants were randomly assigned to each group. Multiple regression analysis, however, suggested that self-initiated action affected the satisfaction even when the preference for the 10 chocolates was controlled.

Detailed analysis of how many chocolates the participants actually chose among the top five favorite chocolates showed that self-initiated action did not affect their choice. The choice analysis indicated no difference in the choice between the groups, but only in satisfaction. This difference between the result of the choice and satisfaction implies that the difference in satisfaction was not due to the choice but to the self-initiated action produced by the choice architecture.

### Experiment 2

In Experiment 1, the result of the multiple regression analysis showed that the preference for the 10 chocolates affected satisfaction as well as the self-initiated action caused by the choice architecture did. This result might have been affected by the survey form. Since Experiment 1 was conducted in the form of a Web survey, we made a hypothetical experimental setting where participants imagined getting the chocolates. Since it was apparently difficult for participants to imagine actually receiving the chocolates, they might have assumed their satisfaction with the five chocolates based on their preferences. For example, if they have a high (or low) preference for chocolates, they might have guessed that their satisfaction with the five chocolates was high (or low). For this reason, in Experiment 1, we might not have measured the actual effect of the self-initiated action. Thus, we examined how the self-initiated action affected the satisfaction when participants could actually receive the chocolates in Experiment 2.

In Experiment 2, we also examined participants’ decision processes of choosing the five chocolates that they could receive. Shafir (1993) showed that decision-makers selectively gather goal-consistent information. For example, when a choice task is given, people easily collect information associated with the positive features, but they have difficulty in collecting negative ones. Contrariwise, when a rejection task is given, people gather information that relates to the negative features well, but they cannot collect sufficient positive information. This phenomenon is known as attentional bias (Shafir, 1993). Given that receiving the five chocolates is regarded as a reward for participants, when participants select five chocolates that they can in fact receive, they may feel task difficulty and greater cognitive conflict in the give-up group than in the choose group. Because of attentional biases, in one study, higher levels of anxiety were associated with faster responses to threat words than neutral words (Mogg et al., 1996). This study indicated that the choice time of the target is associated with psychological feelings. Since the five chocolates are regarded as a reward for participants, they may have a positive feeling for the chocolates and the choice time for five chocolates may be faster in the choose group than in the give-up group. Thus, we predicted that a comparison of choice times between the two groups would be one index of task difficulty and cognitive conflict. Moreover, it is well known that the number of microslips increases as the task becomes more difficult (Reed et al., 2009). Microslips refer to non-smooth hand movements in the course of performing habitual actions (Reed et al., 2009). For example, in the task of making coffee, if a spoon is prepared with a fork at the same time, the number of microslips will be larger than when only a spoon is prepared because participants feel cognitive conflict over using the spoon or the fork to stir their coffee. In addition to comparing the choice times between the groups, we considered microslips effective for measuring cognitive conflict in detail. For these reasons, we took a video when the participants chose five chocolates. Using the data for choice time and microslips, we tried to clarify the cognitive conflict when participants selected five chocolates that they can receive.

#### Participants

We recruited 48 students (*M*_*age*_ = 19.79, *SD*_*age*_ = 2.48, *Men* = 33, and *Women* = 15) from the University of Tokyo and assigned them randomly into either the choose group or give-up group. Experiment 1 found that the effect size of the framing effect generated by the self-initiated action was medium (*d* = 0.46). Cohen (1988) proposed the interpretation of effect size *d* as follows: small effect size: *d* = 0.20, medium effect size: *d* = 0.50, large effect size: *d* = 0.80. Given that participants would actually obtain the chocolates in Experiment 2, we expected that the effect size would be larger in this behavioral experiment than in the online one. Thus, we set a larger effect size (*d* = 0.80) for the second experiment. We used a one-tailed analysis as in Experiment 1 because we had hypothesized that the choose group would be more satisfied with the five chocolates than the give-up group. We used the program G^∗^Power (Version 3.1.9.3; Faul et al., 2007), which indicated that a sample size of 21 participants was necessary for this experiment to have a detection power of 80% for large effects (*d* = 0.80); α was set at 0.05. Accordingly, we recruited around 21 participants for each group.

#### Task, Stimulus, and Procedure

**FIGURE 6:**
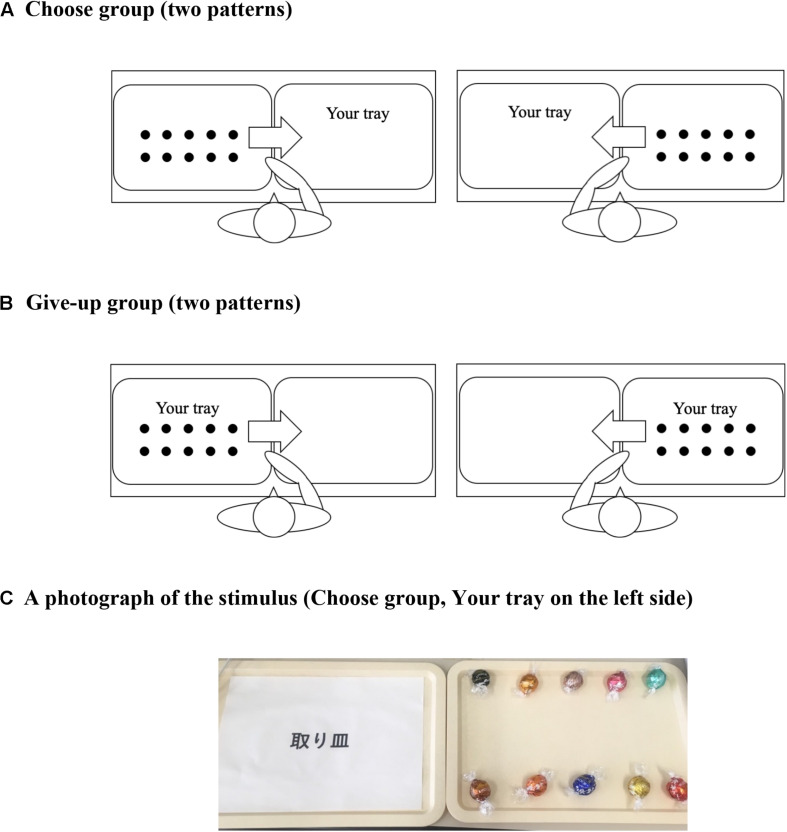
Experimental scenes depicting the choice of five chocolates. The 10 black circles represent chocolates, and the position of the chocolates is the same as in Figure 1A. Panel **(A)** shows the placement for the choose group, while panel **(B)** shows that for the give-up group. Panel **(C)** is an example of the stimulus that was actually used in Experiment 2.

The task and design of Experiment 2 were basically the same as those of Experiment 1. First, participants did irrelevant tasks for 50 min (the task was the same as in Experiment 1). In Experiment 2, participants received five chocolates and 1,000 Yen (approximately US$9.4) as a reward for their participation. To ensure a task length commensurate with an amount of rewards comparable to that in Experiment 1, we asked participants to perform irrelevant tasks for 50 min. Participants were asked their preferences for the 10 types of chocolates after they had finished the irrelevant tasks. The stimuli were the same as shown in Experiment 1. Participants were then asked to select five chocolates as a reward for answering the irrelevant tasks. The original position of the 10 chocolates differed by group (Figures 6A,B). Figure 6C is a photograph of the stimuli actually used in Experiment 2. As shown in Figures 6A,B, “your tray” (i.e., the tray where participants were asked to put the chocolates they wanted to receive) was randomly positioned to the right or left for each participant. The different default position of the 10 chocolates induced different self-initiated actions (choose or give-up). After moving five chocolates, the participants in fact received the five chocolates that they had selected. They were then asked their satisfaction with the five chocolates that they selected as in the same method of Experiment 1. During participants’ choices, we took a video and measured the choice time and the number of their microslips. Before their choices started, they were informed that they were being video-recorded.

#### Results

**FIGURE 7:**
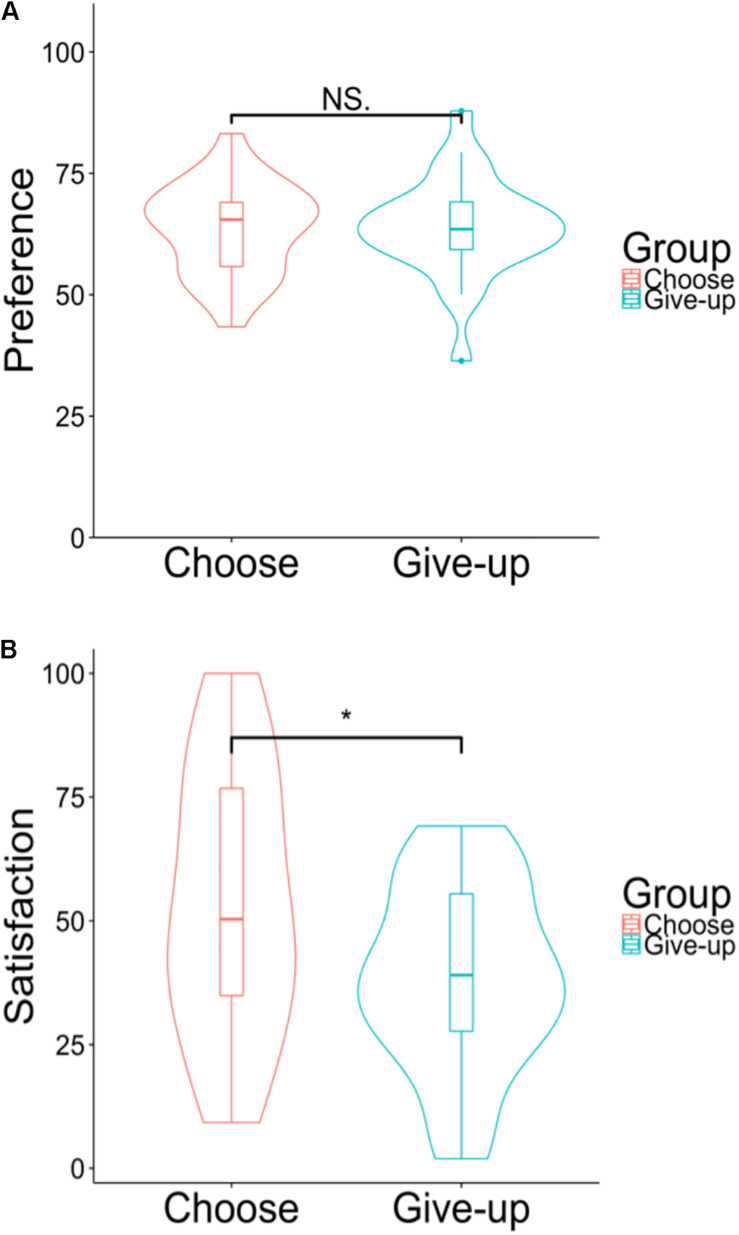
**(A)** Violin plots of the preference for the 10 chocolates. **(B)** Violin plots of the satisfaction with the five chocolates. *NS p* > 0.05, *p < 0.05.

Figure 7 shows the results for the preferences for the 10 chocolates and the satisfaction ratings for the five chocolates. In preferences for the 10 chocolates, there was no significant difference between the choose group (*Mean* = 63.5, *SD* = 9.42) and the give-up group [*Mean* = 64.0, *SD* = 10.40; *t*(46) = –0.15, *p* = 0.881, and *d* = –0.05]. On the other hand, the satisfaction with the five chocolates in the choose group (*Mean* = 53.6, *SD* = 26.90) was significantly higher than that in the give-up group [*Mean* = 38.7, *SD* = 18.47; *t*(46) = 2.23, *p* = 0.029, and *d* = 0.65].

**FIGURE 8:**
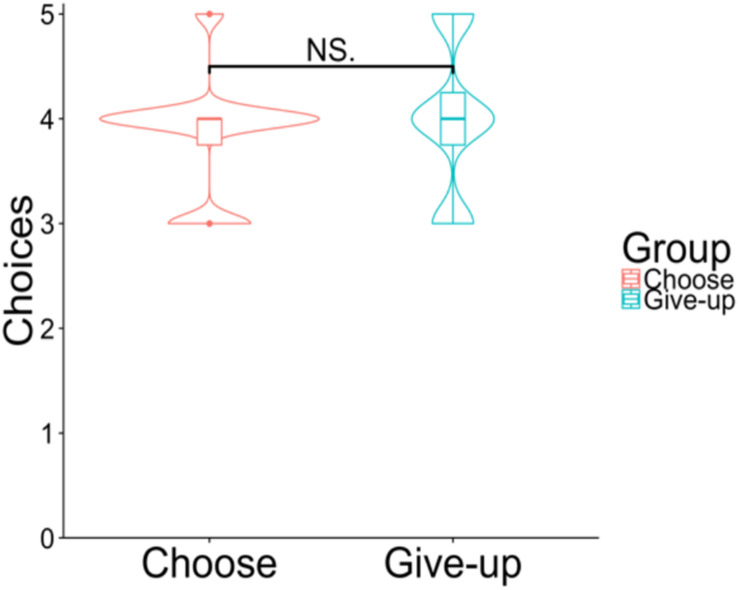
Violin plots of the choices. The *y*-axis shows how many chocolates the participants selected among those with top five preferences. *NS p* > 0.05.

Figure 8 shows the distribution of how many chocolates the participants actually chose from among their five most favorite chocolates; there was no significant difference between the choose group (*Mean* = 3.83, *SD* = 0.95) and give-up group [*Mean* = 4.00, *SD* = 1.17; *t*(43) = –0.89, *p* = 0.378, and *d* = –0.30].

**FIGURE 9:**
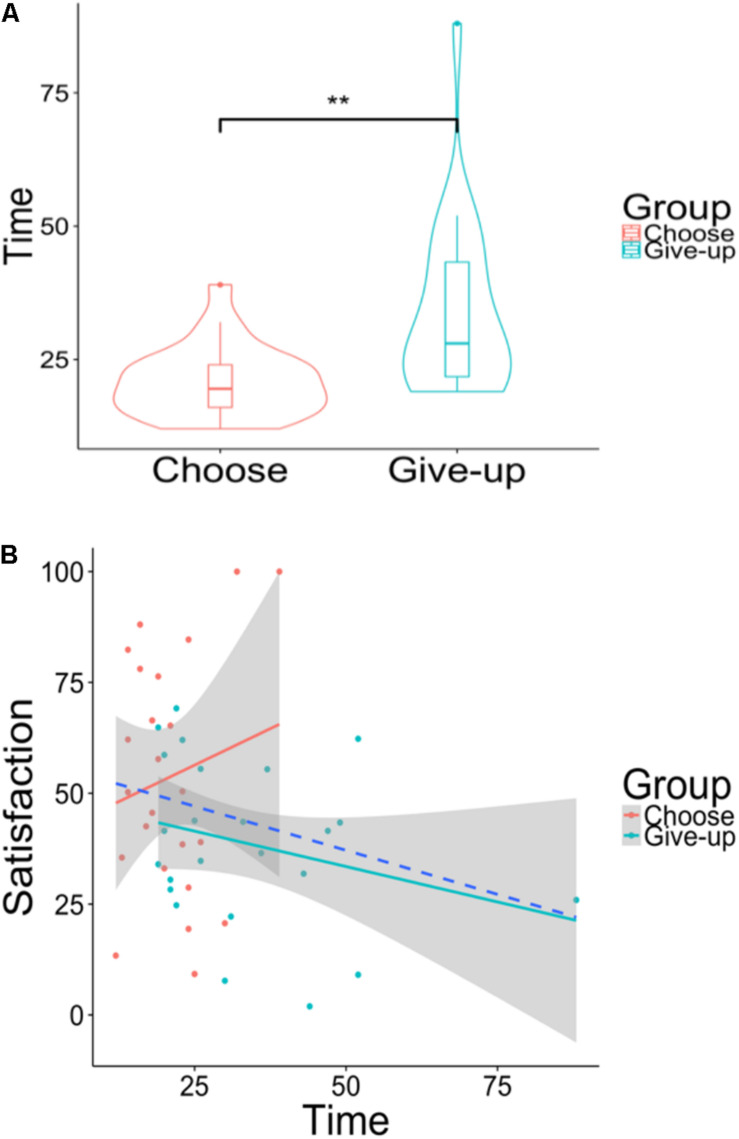
**(A)** Violin plots of the choice time (s) for selecting five chocolates. **(B)** Scatter plot of the choice time when choosing five chocolates versus the satisfaction ratings of the five chocolates. The area of shaded regions shows 95% confidence interval. The dashed blue line shows a negative correlation between the choice time when choosing five chocolates and the satisfaction ratings of the five chocolates when the data of the two groups were gathered into one. **p < 0.01.

Figure 9A shows the participants’ choice time of five chocolates. The choice time was significantly shorter in the choose group (*M*_*sec*_ = 20.87, *SD*_*sec*_ = 6.54) than in the give-up group [*M*_*sec*_ = 33.58, *SD*_*sec*_ = 16.11; *t*(30) = –3.57, *p* < 0.01, and *d* = –1.94]. The Pearson’s correlation coefficient between the participants’ choice time and the satisfaction ratings of the five chocolates when the two groups were gathered into one was not significant (*r* = –0.22, *p* = 0.120; Figure 9B) or for the individual groups (choose group, *r* = 0.16, *p* = 0.454; give-up group, *r* = –0.27, and *p* = 0.187).

**FIGURE 10:**
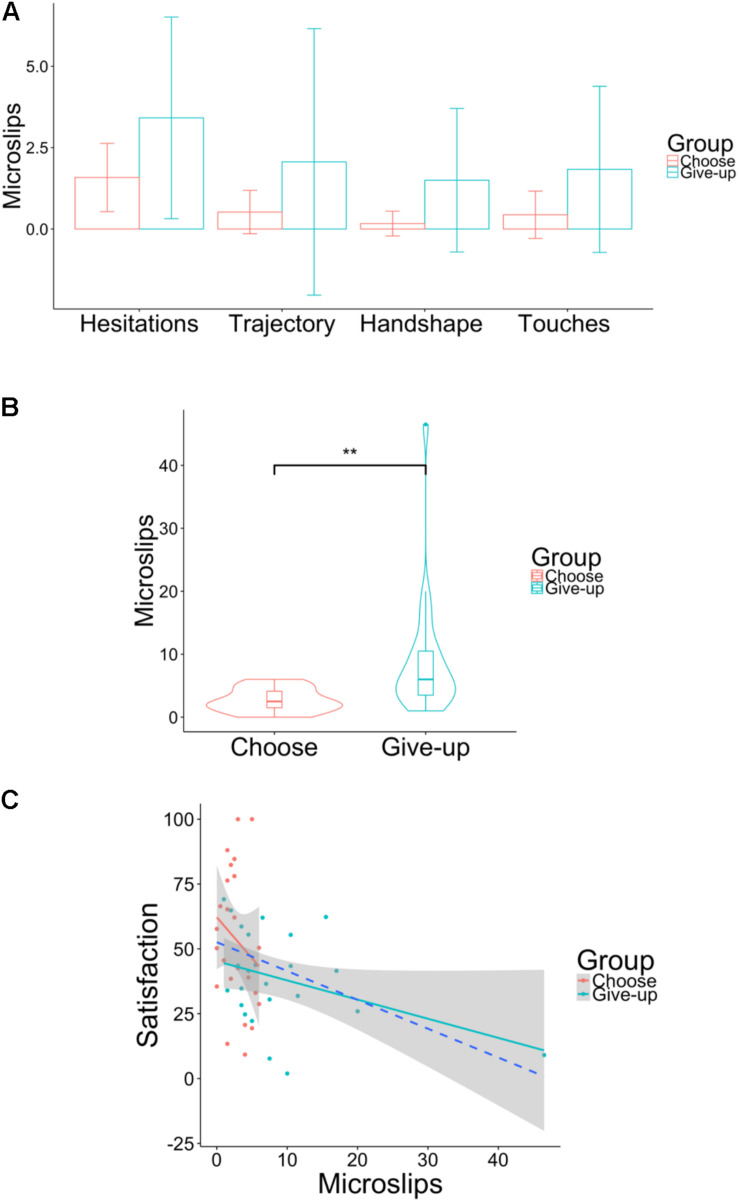
**(A)** Bar graphs of four categories of microslips for each group. The *y*-axis shows the number of microslips. The error bar represents standard deviation. Trajectory means *Trajectory Shifts*, and Handshape means *Handshape Changes.* **(B)** Violin plots of the total number of microslips for the four categories. **(C)** Scatter plot between the total number of microslips including the four categories and the satisfaction with the five chocolates. The area of shaded regions shows 95% confidence interval. The dashed blue line shows the negative correlation between the number of microslips and the satisfaction ratings of the five chocolates when the data of the two groups were gathered into one. **p < 0.01.

Figure 10A shows the number of microslips in the following four categories: *Hesitations*, *Trajectory Shifts*, *Handshape Changes*, and *Touches*, following the classification of Reed et al. (2009). *Hesitations*: Relatively long cessations of movement in the arms and hands. *Trajectory Shifts*: When one or both hands reach in the direction of a target but either stop when they make contact or just prior to making contact and then continue on to a second target. *Handshape Changes*: When one or both hands are shaped to use a target and are moved toward a different object. *Touches*: When participants actually take possession of an object but then relinquish possession without using it. To determine inter-rater reliability, two independent video observers counted the number of microslips, showing a concordance rate (Cohen’s kappa coefficient) of *κ* = 0.304, indicating fair inter-rater reliability (Landis and Koch, 1977). We thus used the average of the data created by the two observers (our data are posted here: https://osf.io/9c2gy). First, in the total number of microslips in the four categories, there was a significant difference between the choose group (*Mean* = 2.70, *SD* = 1.90) and the give-up group [*Mean* = 8.81, *SD* = 9.43; *t*(24) = –3.10, *p* < 0.01, and *d* = –3.2; Figure 10B]. The number of microslips was analyzed using 2 × 4 ANOVAs with the group variable (choose or give-up) and the microslip variable (the four categories above). The analysis indicated a significant main effect of group [*F*(1, 184) = 22.37, *p* < 0.001] as well as of the number of microslips [*F*(3, 184) = 5.14, *p* < 0.01] and no significant effect of interaction [*F*(3, 184) = 0.11, *p* = 0.948]. We performed multiple comparisons on the data above with the Bonferroni correction (Table 2) and found that the number of instances was not significantly different between the two groups for any identical category of microslip. Second, the Pearson’s correlation coefficient was significantly negative between the number of microslips and the satisfaction ratings of the five chocolates when the two groups were combined as one (*r* = –0.34, *p* = 0.017; Figure 10C), but there was no significant correlation between them for either the choose group (*r* = –0.37, *p* = 0.068), or the give-up group (*r* = –0.22, *p* = 0.295).

**TABLE 2:** Results of 2 × 4 ANOVAs with group as between groups (choose or give-up) and four categories of microslips.

	***Hesitations* (choose)**	***Hesitations* (give-up)**	***Trajectory shifts* (choose)**	***Trajectory shifts* (give-up)**	***Touches* (choose)**	***Touches* (give-up)**	***Handshape changes* (choose)**
*Hesitations* (give-up)	*p* = 0.140	–	–	–	–	–	–
*Trajectory shifts* (choose)	*p* = 1	***p* < 0.001**	–	–	–	–	–
*Trajectory shifts* (give-up)	*p* = 1	*p* = 1	*p* = 0.501	–	–	–	–
*Touches* (choose)	*p* = 0.822	***p* < 0.001**	*p* = 1	*p* = 0.104	–	–	–
*Touches* (give-up)	*p* = 1	*p* = 0.094	*p* = 1	*p* = 1	*p* = 1	–	–
*Handshape changes* (choose)	*p* = 1	***p* < 0.001**	*p* = 1	*p* = 0.354	*p* = 1	*p* = 1	–
*Handshape changes* (give-up)	*p* = 1	*p* = 0.421	*p* = 1	*p* = 1	*p* = 0.296	*p* = 1	*p* = 0.890

*Bold font indicates p < 0.05.*

Furthermore, we examined the results in detail using regression analyses in which a dependent variable (rating of satisfaction, choice time, or the number of microslips) was regressed on three independent variables: the group (i.e., choose or give-up, a dummy variable), the position of “your tray” (i.e., right or left, a dummy variable), and the preference for the 10 chocolates (i.e., preference for the 10 chocolates). Tables 3–5 show results of these regression analyses, which indicate that only the group was significant.

**TABLE 3:** Results of multiple regression analysis of satisfaction with the rewards by three factors.

**Relevant factors**	**β**	***SE***	***t*-value**	***p*-value**
Intercept	57.561	22.517	2.556	0.014*
Group (choose or give-up)	–14.000	6.776	–2.066	0.044*
Position of participants’ trays (right or left)	–7.279	6.899	–1.055	0.297
Preference for the 10 chocolates	–0.018	0.349	–0.054	0.957

*p < 0.05.

**TABLE 4:** Results of multiple regression analysis of the choice time by three factors.

**Relevant factors**	**β**	***SE***	***t*-value**	***p*-value**
Intercept	42.102	11.432	3.68	*p* < 0.001***
Group (choose or give-up)	13.463	3.440	3.913	*p* < 0.001***
Position of participants’ trays (right or left)	–4.991	3.502	–1.425	0.161
Preference for the 10 chocolates	–0.304	0.177	–1.715	0.093

***p < 0.001.

**TABLE 5:** Results of multiple regression analysis of the number of microslips by three factors.

**Relevant factors**	**β**	***SE***	***t*-value**	***p*-value**
Intercept	–0.105	6.552	–0.016	0.987
Group (choose or give-up)	6.455	1.972	3.273	0.002**
Position of participants’ trays (right or left)	–3.022	2.007	–1.505	0.139
Preference for the 10 chocolates	0.062	0.101	0.610	0.544

**p < 0.01.

#### Discussion

The results of Experiment 2 showed that satisfaction with the rewards was significantly higher for the choose group than for the give-up group. That is, the self-initiated actions induced by different default positions of the 10 chocolates produced different frames that affected participants’ satisfaction with the five chocolates that they received. The results indicate that although the task set is a logically equivalent linguistic instruction for both groups, the psychological processes differ between them. Multiple regression analysis showed that the preference for the 10 chocolates did not significantly affect the satisfaction rating.

The choice time analyses showed that the choice process was significantly longer for the give-up group than for the choose group. Moreover, the microslip analyses showed that the differences in self-initiated actions caused different numbers of microslips. These results indicated that the difference in self-initiated actions created different cognitive conflicts when participants selected the five chocolates. In other words, giving up five chocolates from the 10 chocolates was more difficult than choosing five chocolates. Previous studies did not show different psychological mechanisms of cognitive conflict depending on the four categories of microslips. We will need to clarify the differences in these four categories in future studies.

## General Discussion

In this study, we conducted two experiments to examine whether the self-initiated action generated by the choice architecture changed the evaluations. The experiments showed that differences in self-initiated actions regarding choice affected the satisfaction rating for the items selected. In particular, participants showed higher satisfaction with the five chocolates they received when their action was “to choose what they wanted” rather than “to give up what they did not want.”

Here, we will discuss the psychological mechanisms why the participants showed different levels of satisfaction with the five chocolates in the two groups, as the psychological choice processes may differ between them. In the choose group, the choosing process can be regarded as “choosing five chocolates.” In the give-up group, the choosing process can be regarded as “giving up (or rejecting) five chocolates.” This difference in the psychological choice processes between “choose” and “give up” might have led to different levels of satisfaction with the five chocolates. Given that the choice time took longer in the give-up group than in the choose group, the tasks were significantly more difficult in the give-up group than in the choose group. The microslip analyses suggest that cognitive conflict was significantly higher in the give-up group than in the choose group. Analyses of choice time and microslips supported a difference in the psychological choice processes of the five chocolates between the groups. Moreover, we found a significant negative correlation between the number of microslips and the satisfaction with the five chocolates. Thus, the results imply that participants’ satisfaction decreases when their cognitive conflict increases.

We point out the relationship between the present findings and the endowment effect (Kahneman et al., 1990). It is well known that when people own an object (e.g., a coffee mug), they attach special value to it and as a result are more likely to retain the object when they feel “ownership” for the object. The framing effect elicited by the self-initiated action is similar to the endowment effect in some respects. For example, participants were ostensibly “given” the 10 chocolates in the give-up group, implying that they might have felt “ownership” of the chocolates. Previous research showed that the more the participants have the opportunity to touch a target, the more they feel ownership of the object (Peck and Shu, 2009; Shu and Peck, 2011). According to these studies, participants might feel “ownership” of the chocolates stronger in the give-up group in Experiment 2 than those in Experiment 1. Since Experiment 1 was conducted in the form of a Web survey, participants could not touch the chocolates, whereas they could in Experiment 2. The difference in the feeling of “ownership” of the chocolates seems to have made a difference in the effect size of the self-initiated action between Experiments 1 and 2.

Previous studies have reported analogous effects to our study. For example, non-verbal information, which means information without language manipulation (Hall et al., 2019), produced framing effects in animals such as wild-caught adult European starlings (Marsh and Kacelnik, 2002) and capuchin monkeys (Lakshminarayanan et al., 2011). These studies showed that the choice architecture sets reference points (the amount of food that the animal expects to get), which influence animals’ choices. Moreover, a previous study showed that self-initiated action changes the frame (Sher and McKenzie, 2006). In previous studies, almost all the methods of generating the framing effects involved manipulating linguistic expressions. The present and previous findings together, however, suggest that other factors than language manipulations can produce framing effects as well, indicating that it is necessary to create a new category of framing effects such as non-verbal framing or self-initiated action-based framing.

We conclude that self-initiated action induced by the choice architecture affected evaluations even when the verbal expressions used were exactly the same. In particular, the difference in the placement of rewards, which required participants to act differently, changed the frame selection and decision-makers’ evaluations. Our findings provide new evidence for ways to generate framing effects besides those where decision-makers are provided with specific frame descriptions.

## Data Availability Statement

All data and materials have been made publicly available via the Open Science Framework and can be accessed at: https://osf.io/9c2gy.

## Ethics Statement

The studies involving human participants were reviewed and approved by the Ethics Review Committee for Experimental Research with Human Subjects at the University of Tokyo’s Graduate School of Arts and Sciences. The patients/participants provided their written informed consent to participate in this study.

## Author Contributions

All authors contributed to the experiment concept and design. Data collection for Experiment 1 was led by HH and YO. Data analyses for Experiment 1 were performed by YO. Researching and data collection, along with data analyses for Experiment 2 were led by YO. YO drafted the manuscript. KU and HH provided critical revisions. All authors approved the final version of the manuscript for submission.

## Conflict of Interest

The authors declare that the research was conducted in the absence of any commercial or financial relationships that could be construed as a potential conflict of interest.

## Footnotes

1 https://insight.rakuten.co.jp/en/

2 http://www.qualtrics.com
